# Differential Activities of Antioxidant Enzymes, Superoxide Dismutase, Peroxidase, and Catalase vis-à-vis Phosphine Resistance in Field Populations of Lesser Grain Borer (*Rhyzopertha dominica*) from India

**DOI:** 10.3390/antiox12020270

**Published:** 2023-01-25

**Authors:** Hagalavadi Vijaykumar Ranjith, Doddachowdappa Sagar, Vinay Kumari Kalia, Anil Dahuja, Sabtharishi Subramanian

**Affiliations:** 1 Division of Entomology, ICAR-Indian Agricultural Research Institute, New Delhi 110012, India; 2 Division of Biochemistry, ICAR-Indian Agricultural Research Institute, New Delhi 110012, India

**Keywords:** bulk grain storage, fumigant, hydrogen peroxide, LC_50_, oxidative stress, oxyradicals, specific activity, toxicity

## Abstract

Susceptibility to phosphine was compared in 15 populations of lesser grain borer (*Rhyzopertha dominica*) collected from grain storage godowns across India. A high level of resistance to phosphine was noticed in *R. dominica* collected from northern India compared to those collected from northeastern regions of India. The median lethal concentration values varied from 0.024 mg/L to 1.991 mg/L, with 1.63 to 82.96-fold resistance compared to laboratory susceptible checks. Antioxidant enzymes have been reported to negate the reactive oxygen species generated upon encountering the fumigant phosphine. Distinct differences in the activity of antioxidant enzymes were noticed in the field populations exposed to phosphine. Peroxidase activity varied between 1.28 and 336.8 nmol H_2_O_2_ reduced/min/mg protein. The superoxide dismutase inhibition rate was between 81.29 and 99.66%, and catalase activity varied between 6.28 and 320.13 nmol H_2_O_2_ reduced/min/mg protein. The findings of our investigation show that the activities of peroxidase and superoxide dismutase are positively linked (*p* < 0.01) with an increase in resistance ratios, whereas catalase was found to have a negative association with resistance to phosphine. The reported results elucidate the differential activities of principal antioxidant enzymes in scavenging the oxyradicals (O_2_^•−^, H_2_O_2,_^•^OH) associated with tolerance to phosphine in *R. dominica.*

## 1. Introduction

Among the several stored grain pests, *Rhyzopertha dominica*, commonly referred to as lesser grain borer, is a primary pest of economic importance. Sound grains of crops such as wheat and paddy are the food and potential breeding substrates of this pest [1,2]. Grain infestation of staple cereals, viz., wheat and rice, by this pest in bulk grain storage threatens food security both nationally and globally [3]. As internal feeders, both grubs and adults of lesser grain borer can scrape wheat kernels to the pericarp and continue to feed on the endosperm and germ [4]. Entire grain lots are sometimes rejected and deemed unfit for human consumption in cases of severe infestation. The frass contains droppings such as exuviae, feces, and immature fragments, affecting the end-use quality and therefore consumer acceptance [5]. Several authors have estimates of losses due to infestation by *R. dominica*, varying from 17% kernel loss [4] to 10 to and 23% weight loss of wheat kernels [6,7]. Adult male and female *R. dominica* have a mean longevity of 26 and 17 weeks, respectively, and can cause a sustained loss of stored wheat grains due to prolonged feeding [8].

Fumigation is the preferred practice for controlling stored grain pests. Aluminum phosphide tablets are regularly administered at a rate of 3 tablets/ton of grain or 14 tablets/28 m^3^ of storage area during peak breeding and infestation stages of insect pests. India stores 60 million tons of grain annually, and phosphine fumigation is carried out to control stored grain pests including *R. dominica*. The prevention of avoidable post-harvest losses of grain due to insect pests can provide an additional supply of food grains [9]. 

Fumigation with phosphine [10] is an efficient and dependable chemical method to manage insect pests, as it effectively kills the major infesting grub/larval stage, as well as resting pupa and emerging adults. Phosphine has been used on a global scale for the protection of stored grain products for over fifty years [11]. Overdependence on phosphine for stored grain pest management has led to control failures [12]. Resistance to phosphine fumigation in storage insect pests was brought to light by a global survey by Champ and Dyte [13]; one among the ten individuals of the collected insect populations was found to be resistant to phosphine according to this survey. There was a higher degree of occurrence of resistance to phosphine in *R. dominica* populations, with 8 of 21 populations exhibiting resistance to phosphine on stored wheat in farms in Oklahoma [14]. Strong resistance to phosphine in *R. dominica* was registered for the first time in the southern Queensland region of Australia in 1997 [15].

The first report of control failure by phosphine fumigation in stored grain insects in India was recorded by Bora and Chahal [16] as early as 1979. Among the insect pests, *T. castaneum*, *R. dominica*, and *Oryzaephilus surinamensis* were found to have developed significant levels of resistance to phosphine in India [17,18]. A phosphine resistance monitoring survey in India revealed 14 *Trogoderma granarium* populations with varying levels of resistance to this fumigant [19]. 

Two major genes are linked to a strong resistance phenotype in *R. dominica*. A genetic study mapped the resistance genes *rph*1 and *rph*2 in *R. dominica* [20]. While each gene is associated with weak resistance independently, the interaction of the two genes induces a strong resistance phenotype. The synergistic interaction of the *rph*1 and *rph*2 genes was found to cause a strong resistance to phosphine in *R. dominica* [21]. The discovery of a core metabolic enzyme, dihydrolipoamide dehydrogenase (DLD), as the *rph*2 gene paved the way to design CAPS (cleaved amplified polymorphic sequence) markers for quick genotyping of insect populations showing resistance to phosphine. This CAPS marker has been widely used for the detection of resistance status in *T. castaneum* and *R. dominica* populations collected from godowns in various countries, viz., USA, Australia, and Turkey [22,23,24]. A study by Kaur and colleagues revealed that strong resistance to phosphine was linked to a P49S variant in the *rph*2 gene, which was found to have conserved occurrence in different countries [25]. 

As a respiratory toxin, phosphine affects the redox state in mitochondria and especially complex IV cytochrome c oxidase, inducing the generation of oxyradicals (ROS) such as hydrogen peroxide (H_2_O_2_) and superoxide (O_2_^•−^), which induce oxidative stress in cells, resulting in cell death [26]. There is cumulative evidence of the involvement of ROS with the toxicity of several xenobiotics including metal compounds and pesticides in insects [27]. Synthetic insecticides and bioinsecticides were found to influence the activity of antioxidant enzymes polyphenol oxidase, SOD, peroxidase, and catalase in a bruchid, *Callosobruchus* sp., infesting pulses [28]. The cell membrane was found to be less susceptible to ROS when the insects were homozygous for *rph*1, and fewer ROS were produced when the resistant allele *rph*2 was homozygous. When an insect shows a high level of phosphine resistance (i.e., homozygous to both alleles), the production of ROS is curtailed or it is found to be less susceptible to the ROS. 

Phosphine-induced oxidative damage is regarded as a key mechanism of its toxicity in insects. The toxic action of phosphine invariably affects the metabolic energy production system in mitochondria [29]. Phosphine is also known to disrupt the antioxidant defense system by inducing the activity of superoxide dismutase and reducing the activity of catalase and peroxidase in *R. dominica* [30]. Phosphine fumigation was reported to induce the activity of SOD and to inhibit catalytic activity in insect pests and mice [31]. A differential response of antioxidant enzymes was observed between resistant and susceptible individuals, and increased antioxidant enzyme activity is associated with phosphine tolerance in insects [32]. The responses of main antioxidant enzymes to phosphine fumigation in *Drosophila melanogaster* revealed downregulation of the catalase gene through the signal transduction process [33]. A recent study showed the differential expression of antioxidant genes in susceptible and resistant strains of *R. dominica* [34]. Significant positive correlations of peroxidase and SOD activities were noticed vis-à-vis the resistance ratio in field populations of *T. granarium* collected across North India [19]. 

Although phosphine continues to be used as a sole fumigant in bulk grain storage in India, there is scant information available on the current status of phosphine resistance in major stored grain pests such as *R. dominica* in the country. Hence, the present study was conducted to screen 15 field populations of *R. dominica* collected across northern and northeastern regions of India. We examined the antioxidant enzyme activities in field populations of *R. dominica* upon exposure to phosphine to ascertain the relationship between phosphine toxicity and antioxidant enzymes. The outcome of this study could help manage the growing problem of phosphine resistance and support the development of an effective management strategy for this dreaded pest of food grains.

## 2. Materials and Methods

### 2.1. Test Insect

**Figure 1:**
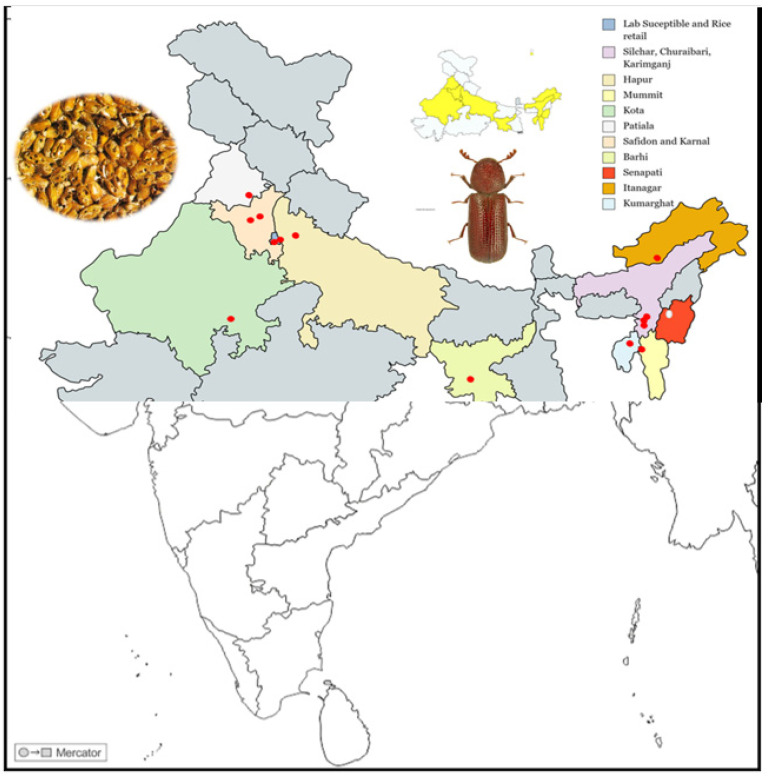
Locations of the *Rhyzopertha dominica* populations that were surveyed and collected in India. The political map of India shows different states bordered by thin lines. The geographical region of each state is marked with colors in the background. The Collection sites are marked with a red dot (•), and the location details are mentioned as legends of this figure in the top-right corner. The map was sourced from http://d-maps.com/carte.php?num_car=4183&lang=en accessed on 11 July 2022 and slightly modified in Microsoft Excel to the current frame.

A total of populations of lesser grain borer (*R. dominica*) were collected from 11 Indian states, viz., Uttar Pradesh, New Delhi, Haryana, Punjab, Rajasthan, Mizoram, Assam, Jharkhand, Manipur, Arunachal Pradesh, and Tripura, during the period of 2019–2020. Among the collected populations, five were collected from northern Indian states in and around Delhi, one from Jharkhand, and eight from the northeastern states, with one maintained in the laboratory to be used as a susceptible population. The details of field collections are presented in Table 1 and Figure 1. Initial populations of approximately 250–500 beetles of *R. dominica* from collection locations were brought to the laboratory and maintained in separate glass jars (1.5 l) containing wheat grains enriched with 5% brewer’s yeast. The laboratory population maintained at the Division of Entomology, IARI, New Delhi, for more than 30 generations without exposure to phosphine gas fumigation was used as the susceptible reference strain. The insects were maintained at a temperature of 30 ± 2 °C and relative humidity of 65 ± 5% RH.

### 2.2. Bioassay

The field populations of *R. dominica* were brought to the laboratory and maintained by diet for two generations. A phosphine bioassay on the populations of *R. dominica* was conducted as per FAO protocol [35]. The discriminatory dose prescribed by the FAO for *R. dominica* was taken into consideration when the doses were selected. Phosphine was generated using aluminum phosphide 56% F tablets immersed in acidified water (5% sulfuric acid) and collected in a glass tube set up for this purpose. Adult beetles (aged 15 days) were placed in thermostable plastic vials and exposed to a series of phosphine concentrations (0.01, 0.03, 0.06, 0.1, 0.5, 1.0, 1.5, 2.0, and 2.5 mg/L) within air-tight desiccators. Each population was subjected to a control treatment by injecting air into the desiccator instead of phosphine.

Each plastic vial contained 30 individuals, with three vials per desiccator. The vials were wrapped in a muslin cloth and labeled appropriately. To attain the desired concentration, the measured volume of phosphine gas was then administered using air-tight microliter syringes (Hamilton, Germany) via a septum in the lid of each desiccator. The vials were taken out of the desiccators and fed after being exposed for 24 h; the mortality readings were taken after seven days. Inactive (moribund) beetles were considered dead. 

## 3. Phosphine Treatment and Determination of Resistance Ratio

Adult beetles chosen from the F_1_ generation comprising the field populations were used for the study. Two-week-old adult beetles were exposed for 24 h to determine the LC_50_ concentration of phosphine. The mortality data were used for probit analysis to determine LC_50_ values [35]. A Laboratory susceptible population (Pusa) was maintained without any selection pressure (phosphine exposure) for at least thirty generations. This strain was used as a susceptible check to calculate the resistance ratios in collected populations. The resistance ratios of the field populations were computed by dividing the LC_50_ value of the respective population by the LC_50_ value of the susceptible population and multiplied by one hundred. 

## 4. Enzyme Assays

The enzyme activities of superoxide dismutase, catalase, and peroxidase were measured in the *R. dominica* field populations. Beetles surviving after 24 h of phosphine exposure to LC_50_ concentrations were used for enzyme assays. The survivor adult beetles were homogenized in microcentrifuge tubes (1.5 mL) containing 100 µL of 50 mM phosphate buffer (pH 7.0). The homogenates were centrifuged at 16,000 rpm for 10 min in a refrigerated centrifuge (Eppendorf, Germany) at 2–6 °C. The supernatants were used as enzyme extracts for assays. The specific activity of (catalase and peroxidase) was calculated by dividing the activity of the respective antioxidant enzyme by the protein content of the sample as mentioned below.

$$\mathrm{nmol}/\min/\mathrm{mg}\;\mathrm{protein}=\frac{\mathrm{Change}\;\mathrm{in}\;\mathrm{OD}/\min\;\times \;\mathrm{total}\;\mathrm{reaction}\;\mathrm{volume}\;\left(\mathrm{mL}\right)\times 1000}{\mathrm{Extinction}\;\mathrm{coefficient}\;\times \;\mathrm{path}\;\mathrm{length}\;\times \;\mathrm{protein}\;\left(\mathrm{mg}\right)}$$

## 5. Catalase Assay

In microplate wells, catalase activity was assayed using a mixture of 50 µL of 50 mM phosphate buffer (pH 7), 50 µL of freshly generated 20 mM H_2_O_2_, and 20 µL of enzyme extract. A microplate reader spectrophotometer was used to measure the reduction in absorbance induced by H_2_O_2_ breakdown for 3 min at 240 nm. Catalase activity was measured using a 39.4 mM^−1^ cm^−1^ extinction coefficient and reported as nmol of H_2_O_2_ degraded per minute per milligram of protein [36]. Blank solutions devoid of enzyme extract were used to control the non-enzymatic processes.

## 6. Peroxidase Assay

Peroxidase activity was determined in microplate wells using a cocktail of 100 µL of 50 mM phosphate buffer (pH 7), 60 µL of guaiacol, 20 µL of enzyme extract, and 60 µL of a freshly prepared 0.1 M H_2_O_2_ [37]. A 96-well microplate reader was used to quantify the rise in absorbance induced by accelerated substrate oxidation in the presence of H_2_O_2_. An extinction coefficient of 26.6 mM^−1^ cm^−1^ was used to calculate peroxidase activity as nmol per minute per milligram of protein. Blank solutions devoid of enzyme extract were used to control the non-enzymatic processes.

## 7. Superoxide Dismutase Assay

Superoxide dismutase (SOD) activity was measured using a commercially available test kit (catalogue no:19610-1KT-F) (Sigma-Aldrich Chemical, St. Louis, MI, USA) according to the manufacturer’s protocol. A SOD kit determines the inhibition activity of SOD by measuring the decrease in color development at 440 nm. The absorbance at 440 nm was measured using a microplate reader (Medispec, Gaithersburg, MD, USA), and the SOD activity was expressed as % inhibition rate.

## 8. Protein Estimation

Protein content was determined by following the method described in [38], with bovine serum albumin (BSA) as the protein standard. Absorbance at 595 nm was measured using a microplate reader spectrophotometer (Medispec, USA).

## 9. Statistical Analysis

Log-dose probit mortality data were analyzed using the Polo Plus 2.0 program [39] to generate regression lines, LC_50_ values, and fiducial limits. The corrected mortality was used for probit analysis. To test whether the difference between treatments was statistically significant, an analysis of variance (*p* < 0.05) was computed, and the treatment means were separated using Duncan’s multiple range test using SPSS software. Pearson correlation was tested using ‘R’ software (R 2.1.9) among the dependent variables—catalase, peroxidase, and SOD—with LC_50_ as the independent variable. Principal component analysis (PCA) was performed, and a biplot was generated to substantiate the interrelation between LC_50_ values and the antioxidant enzyme activities of *R*. *dominica* field populations. The XL STAT 2022 package was used for PCA.

## 10. Results

### 10.1. Susceptibility of Field-Collected Populations to Phosphine

The mortality values expected by probit analysis did not differ significantly from the observed bioassay values; hence, the probit fit was considered appropriate. According to the results of probit analysis (Table 2), the susceptibility of the populations (LC_50_) ranged from 0.024 to 1.991 mg/L from the laboratory susceptible population (Pusa) (designated as “laboratory susceptible check”) to the Safidon population (“resistant check”), respectively. The laboratory susceptible population (Pusa) was the most susceptible to phosphine gas, with LC_50_ values of at least 0.024 mg/L. Most of the field populations of *R. dominica* collected from northeastern India, such as Mammit, Silchar, Kumarghat, Senapati, Churaibari, and Itanagar, were relatively more susceptible to phosphine, with LC_50_ values of 0.039, 0.044, 0.059, 0.065, 0.085, and 0.092, respectively; whereas *R. dominica* from Barhi (Jharkhand) and Karimganj (Assam) showed moderate susceptibility, with LC_50_ values of 1.207 mg/L and 1.146 mg/L, respectively; field populations from Delhi, Kota, Karnal, Hapur, and Patiala located in northern India showed moderate to high resistance to phosphine, with LC_50_ values ranging from 1.221 to 1.708 mg/L; and the *R. dominica* population from Safidon (Haryana) was the least susceptible to phosphine, with the highest LC_50_ value (1.991 mg/L). When compared to the laboratory susceptible population (Pusa), the resistance ratios of the field populations at LC_50_ ranged from 1.63- (Mammit) to 82.96- (Safidon)-fold across the tested populations. A calculated χ2 value less than the χ2 value obtained from the Table 2 (table χ2 = 7.815 for 3 degrees of freedom) at a 5% significance level indicates homogeneity. Therefore, investigated populations are homogenous (Table 2).

### 10.2. The Specific Activity of Antioxidant Enzymes in R. dominica

#### Peroxidase Activity

Significant differences (F = 1047; *p* < 0.05) in peroxidase activities were observed in *R. dominica* field populations, with the specific activities ranging from 1.281 to 336.8 nmol H_2_O_2_ reduced min^−1^mg^−1^ of peroxidase. Among the populations, Silchar exhibited the least activity of 1.281 nmol H_2_O_2_ reduced min^−1^ mg^−1^; field populations from northeastern India exhibited relatively lower specific activity (Kumarghat, 1.805; Churaibari, 2.104; Barhi, 2.583; lab susceptible, 5.35; Itanagar, 10.43 nmol H_2_O_2_ reduced min^−1^mg^−1^, respectively). It is interesting to note that northern Indian field populations, such as Kota, Karnal, and Patiala, showing more tolerance to phosphine exhibited relatively higher specific activity of peroxidase (Table 3). *R. dominica* collected from Safidon (Haryana), which was the least susceptible population to phosphine, exhibited the highest peroxidase activity of 336.8 nmol H_2_O_2_ reduced min^−1^mg^−1^.

### 10.3. Superoxide Dismutase Activity

The inhibition of superoxide dismutase varied between 81.29 and 99.66 % among the field populations of *R. dominica*. Whereas the laboratory susceptible population (Pusa) recorded the lowest inhibition rate of SOD activity (81.29 percent), the highest inhibition of SOD (99.66 percent inhibition rate) was recorded in the Safidon population (F = 299.29, *p* < 0.05). SOD inhibition was observed to follow a similar trend to that of peroxidase, whereby the field populations from the northeastern states of India showed relatively lower SOD inhibition (Silchar, 81.93%; Churaibari, 83.0%; Kumarghat, 83.4%; Barhi, 85.75%; Itanagar, 90.71%; Karimganj, 92.17%), relatively higher rates of SOD inhibition (97.013% to 99.66 1%) were observed in the northern Indian populations. With decreasing susceptibility to phosphine, an increase in inhibition of SOD activity was noticed (Table 3).

### 10.4. Catalase Activity

The specific activity of catalase varied significantly among populations of *R. dominica* (F = 674.25; *p* < 0.05), with values ranging from 6.287 to 320.13 nmol H_2_O_2_ reduced min^−1^ mg^−1^ (Table 3) in the Silchar and laboratory susceptible populations of *R. dominica*, respectively. The field populations that are relatively more susceptible to phosphine such as Churaibari, Senapati, and Kumarghat in northeastern India exhibited significantly higher catalase activities of 35.22, 46.33, and 59.06 nmol H_2_O_2_ reduced min^−1^mg^−1^, respectively, whereas populations tolerant to phosphine, viz., Patiala, Safidon, Karnal, Hapur, and Kota, recorded lower catalase activities of 7.701, 7.764, 8.771, 11.104, and 13.11 nmol H_2_O_2_ reduced min^−1^mg^−1^, respectively.

### 10.5. Pearson Correlation and PCA of Antioxidant Enzyme Activities

**Figure 2:**
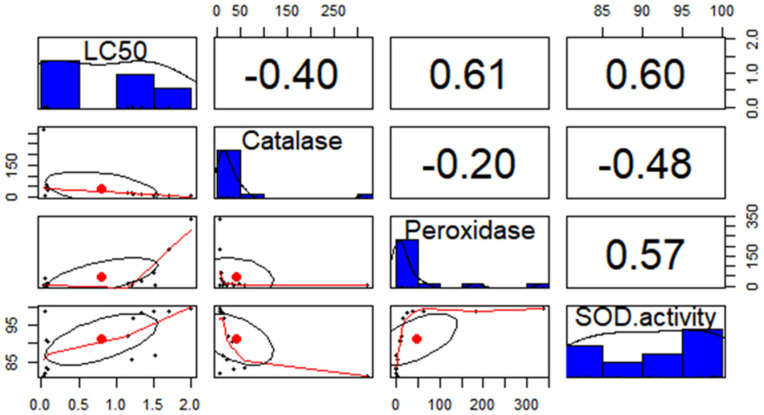
Correlation of LC_50_ with the antioxidant enzymes—catalase, peroxidase, and SOD—in *Rhyzopertha dominica* populations. In this figure, LC_50_ and each of the antioxidant enzymes is marked in the histogram on the diagonal, the upper triangular matrix depicts Pearson correlation, and the lower triangular matrix shows a bivariate scatter plot with a fitted line.

This correlation analysis was carried out to measure the strength of antioxidant enzyme (peroxidase, SOD, and catalase) activity vis-à-vis resistance ratios of phosphine in the field populations of *R. dominica* (Table 4 and Figure 2). Whereas the activities of peroxidase and SOD inhibition were positively correlated with resistance ratios of phosphine, a strong negative correlation was observed concerning catalase activity vis-à-vis phosphine resistance. The values of Pearson correlation coefficients (Figure 2), peroxidase (+0.61), and SOD (+0.60) were similar, exhibiting a medium uphill trend, whereas the specific activity of catalase was found to show a weak downhill trend (−0.40) according to LC_50_ values of phosphine recorded in the studied field populations. Multiple regression analysis using LC_50_ as an independent variable and the enzymes (catalase, peroxidase, and SOD) as a dependent variable revealed that among the antioxidant enzymes, SOD showed the strongest response, with the highest “b” value of 3.151, followed by peroxidase (0.205) and the catalase enzymes (1.34).

**Figure 3:**
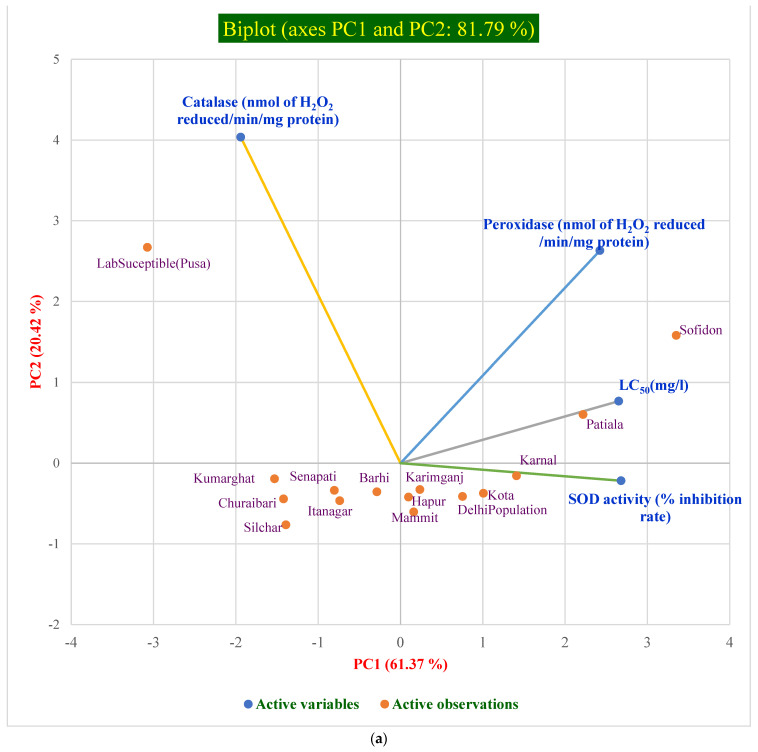

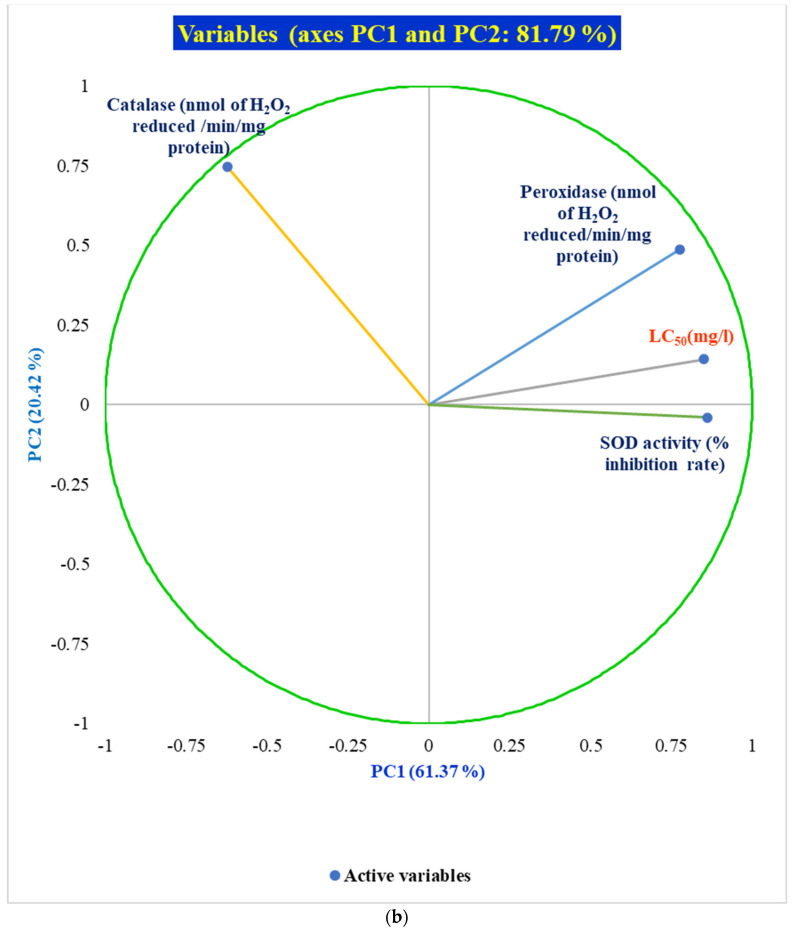
(**a**) Visualization of Pearson correlation showing the influence of antioxidant enzymes on the LC_50_ of the populations used in this study. (**b**) Visualization of the four variables of each of the antioxidant enzymes used in this study, viz., catalase, peroxidase, SOD, and LC_50_. In the above biplot of the principal component analysis (PCA), PC1 and PC2 are represented on the horizontal and vertical axes, with a cumulative variance of 61.37% and 20.42%, respectively. The Eigenvalues of PC1 and PC2 are 2.45 and 0.82, considered as first and second factor, respectively; blue dots (•) in Figure 3a,b represent the four active variables (referring to LC_50_ and each of the antioxidant enzymes, viz., catalase, peroxidase, and SOD), and orange dots (•) in Figure 3a represent the 15 active observations referring to different populations in the study. The distance of the variables from the center indicates the magnitude of influence.

PCA analysis was performed using four variables (catalase, peroxidase, SOD, and LC_50_), and two factors, viz., PC1 and PC2, with the highest Eigenvalues of 2.45 and 0.82, respectively, were chosen, as they accounted 61.37% and 20.42% of variance, respectively (Supplementary Materials Table S1). The biplot graph (Figure 3a) shows that peroxidase was positively related with PC1 and PC2, whereas SOD was positively related to PC2 and negatively related to PC1. The influence of LC_50_ was significantly high on peroxidase and SOD activities, whereas it had a very strong negative correlation with catalase (Figure 3b).

The biplot depicts that the populations showing high resistance to phosphine such as Safidon (LC_50_ = 1.991 mg/L), Patiala (LC_50_ = 1.708 mg/L), and Karnal (LC_50_ = 1.498 mg/L) were distributed together in the positive region of the biplot, in correspondence with peroxidase and SOD activity, and the populations from northeastern India and the lab susceptible population (Pusa) (LC_50_ = 0.024 mg/L) were grouped together.

## 11. Discussion

The international acceptance of grains fumigated with phosphine, owing to its cost-effectiveness and the lack of availability of suitable alternative fumigants, has led to over-reliance on phosphine. The long-term usage of phosphine fumigation and lack of adoption of good fumigation practices have resulted in the emergence of phosphine resistance in several storage insect pests worldwide. India has reported several cases of resistance development in stored grain pests such as *T. castaneum* and *R. dominica* over the years. Strong resistance to phosphine in *R. dominica* had been recorded earlier [17]. Subsequent studies on screening using FAO-recommended discriminatory doses revealed that the frequency of resistance was as high as 100 and 95 % for *T. castaneum* and *R. dominica*, respectively [40]. The occurrence of phosphine resistance is governed by resistance alleles present within the population and also due to the selection pressure created by phosphine fumigation. An *R. dominica* strain (IRD_Mdu_) from Madurai in southern India was reported to be strongly resistant (LC_50_ = 2.2 mg/L), with a resistance ratio as high as 1283-fold relative to a reference strain (QRD_569_) from Australia [25]. A comparative analysis with an Australian susceptible reference strain (QRD_14_) showed that the least susceptible population recorded in this study, Safidon, Haryana (LC_50_ = 1.991 mg/L), can be considered strongly resistant, with a comparable resistance ratio of 1144-fold. Furthermore, eight of the fifteen *R. dominica* populations had LC_50_ values higher than 1 mg/L, which is equivalent to 658-fold resistance against the reference strain, QRD_14_, from Queensland, Australia.

Whereas most of the *R. dominica* populations collected from northern India are relatively more resistant to phosphine, the bulk of the northeastern populations still show susceptibility to phosphine with lower LC_50_ values. This may be due to the storage of food grains for a shorter period and frequent replenishment of grain stocks, which might have led to less frequent fumigation, thereby minimizing resistance development to a lesser extent in northeastern India. Strong resistance to phosphine was previously recorded in *T. castaneum* populations collected across bulk grain storage facilities in northern Indian states such as Uttar Pradesh, Punjab, Haryana, Madhya Pradesh, and Rajasthan [18]. Detailed molecular analysis revealed that mutations in the *rph*2 locus of the dihydrolipoamide dehydrogenase (DLD) were linked to strong resistance to phosphine in *T. castaneum* and *R. dominica* [41]. An earlier study [25] documented the prevalence of resistance to phosphine in *R. dominica* collected from southern India. Results of our study reiterate that *R. dominica* populations collected from storage godowns across northern India also show strong resistance to phosphine.

Although DLD has been linked to phosphine resistance in insects, various studies have demonstrated the relevance of respiration and fumigant uptake, as well as the involvement of mitochondrial enzymes in phosphine toxicity [42,43]. Because phosphine toxicity is based on oxidative respiration, the involvement of energy metabolism in phosphine toxicity/resistance was hypothesized [30,42,43,44]. Phosphine’s action of generating oxygen-derived free radicals, leading to cellular damage, appears to be one of the important factors associated with phosphine toxicity in insects. Phosphine has been shown to cause oxidative damage to macromolecules in insects [29]. Antioxidant enzymes such as SOD, catalase, and peroxidase regulate phosphine-induced oxidative stress. Previous research showed that phosphine affects the antioxidant system by boosting SOD activity while temporally suppressing catalase and peroxidase activity in a dose-dependent manner [30]. In a few instances, resistance to phosphine under hypoxic conditions has been attributed to the suppression of oxidative metabolism [45,46].

Superoxide dismutase (SOD) catalyzes the dismutation of the superoxide anion (O2-) into hydrogen peroxide and molecular oxygen. As color development is related to the amount of superoxide anions, SOD inhibition was estimated by recording the decrease in color development at 440 nm. After phosphine treatment, susceptible insects showed higher inhibition of SOD, peroxidase, and catalase than resistant insects [46]. An earlier study [19] demonstrated that SOD and peroxidase activities were higher in the susceptible population, and catalase activity was non-significant vis-à-vis resistance to phosphine in a *T*. *granarium* population. Our results also show that SOD and peroxidase activities share a similar trend in *R. dominica* in the resistant and susceptible populations. Increased SOD activity might be attributed to increased synthesis, decreased breakdown, and enhancement by inducers (H_2_O_2_ itself acts so)—or all these factors.

A significant positive correlation (+0.689 at *p* < 0.01) of SOD activity and a negative correlation of catalase activity (−0.707 at *p* <0.01) with phosphine resistance ratios were observed in our study (Table 4). Regression analysis of LC_50_ values of different *R. dominica* populations vis-à-vis enzyme activities (Figure 2) similarly reaffirmed that SOD (+ 0.61) and peroxidase (+0.60) had a significant positive correlation, whereas catalase was strongly negatively correlated (−0.40) with the phosphine toxicity.

Our results are consistent with previous reports [20,26,32]. The increase in O_2_^•−^ generation due to phosphine toxicity could have led to the cumulative accumulation of hydrogen peroxide (H_2_O_2_) and the subsequent production of the (HO^•^) hydroxyl radical, a potent oxidizing agent. The elevated levels of antioxidant enzyme activity in resistant populations might have acted as a detoxifying mechanism, enabling improved survival of the resistant insects. Bolter and colleagues [46] reported significantly higher activity of catalase (62%) in susceptible insects than in resistant insects after phosphine exposure. Similar to their studies, our results also show significantly higher (4123-fold) catalase activity in the susceptible check (Pusa) compared to the Safidon population, showing strong resistance to phosphine.

PCA biplot analysis was used to partition distinct differences between the resistant and susceptible populations of *R. dominica.* Our analysis revealed that phosphine resistance levels could be linked positively to the peroxidase and SOD activities (Figure 3a,b) and negatively with catalase activity. The greater inhibition of peroxidase in susceptible populations could have led to the buildup of hydrogen peroxide, resulting in oxidative stress in the cells. The reduced inhibition of these enzymes (peroxidase and SOD), together with a stronger base level in resistant strains, supports a phosphine resistance mechanism.

The increased activities of antioxidant enzymes such as SOD and peroxidases may act as a defense mechanism to nullify the harmful effect of toxic oxyradicals induced by phosphine toxicity. Recent studies [30] using molecular tools demonstrated the inhibition of the catalase gene at the transcriptional level in *D. melanogaster* upon exposure to phosphine treatment. It was inferred that phosphine does not directly inactivate its target enzymes but rather inhibits them by interfering with a complex signal transduction mechanism.

The results of our study provide the current levels of resistance status to phosphine in contemporary populations of *R. dominica* in India. Our study also demonstrates the differential action of three major antioxidant enzymes in negating phosphine toxicity in resistant and susceptible populations of *R. dominica*. Further studies unraveling the underlying molecular mechanisms and variations in mutations of the antioxidant enzymes may shed light on the relationship between structural toxicity and phosphine toxicity in insects.

## Figures and Tables

**Table 1:** Location details of *Rhyzopertha dominica* field populations.

Sl. No	Region	Location	Storage Details	Food Grain	GPS Coordinates
1	Haryana	Safidon	Bulk grain storage	Wheat	29°23′48″ N 76°39′13″ E
2		Karnal	Bulk grain storage	Wheat	29°40′47″ N 76°57′13″ E
3	Punjab	Patiala	Bulk grain storage	Wheat	30°21′25″ N 76°24′59″ E
4	Rajasthan	Kota	Farm storage	Wheat	25°07′48″ N 75°48′34″ E
5	Uttar Pradesh	Hapur	Bulk grain storage	Wheat	28°44′25″ N 77°46′09″ E
6	Delhi	Pusa	Laboratory population	Wheat	28°36′ N 77°13′ E
7		Delhi	Farm storage	Rice	28°37′55″ N 77°08′42″ E
8	Jharkhand	Barhi	Farm storage	Wheat	24°18′11″ N 85°24′48″ E
9	Assam	Karimganj	Farm storage	Wheat	24°52′18″ N 92°22′18″ E
10		Silchar	Farm storage	Rice	24°49′ N 92°48′ E
11		Churaibari	Farm storage	Wheat	24°26′07″ N 92°14′28″ E
12	Manipur	Senapati	Bulk grain storage	Rice	25°11′53″ N 93°59′23″ E
13	Tripura	Kumarghat	Bulk grain storage	Wheat	24°09′49″ N 92°02′18″ E
14	Arunachal Pradesh	Itanagar	Farm storage	Wheat	27°05′ N 93°37′ E
15	Mizoram	Mammit	Bulk grain storage	Rice	23°54′40″ N 92°29′23″ E

This table provides the location details, substratum or host grains of *R. dominica* field populations collected from bulk grain/farm storage godowns in northern and northeastern states of India; the latitudes and longitudes of the collection sites are also mentioned in the last column of the table.

**Table 2:** Toxicity of phosphine in different populations of *Rhyzopertha dominica*.

Sl. No	State	Population	LC_50_ (mg/L)	Fiducial Limit (95%)	Slope ± SE	χ^2^	H	d.f	RR (%)	‘*p*’ Value
1	Haryana	Safidon	1.991	1.555–2.546	4.00 ± 1.28	0.008	0.003	3	82.96	0.999
2	Punjab	Patiala	1.708	1.129–2.124	3.44 ± 1.15	0.311	0.104	3	71.17	0.957
3	Uttar Pradesh	Hapur	1.514	1.029–1.789	4.86 ± 1.43	0.537	0.179	3	63.08	0.910
4	Haryana	Karnal	1.498	0.801–1.871	3.39 ± 1.16	0.543	0.181	3	62.42	0.909
5	Rajasthan	Kota	1.33	0.809–1.783	2.31 ± 0.65	1.029	0.343	3	55.42	0.794
6	Delhi	Delhi	1.221	0.825–1.557	2.59 ± 0.62	2.176	0.725	3	50.88	0.536
7	Jharkhand	Barhi	1.207	0.569–1.484	4.36 ± 1.43	2.016	0.672	3	50.29	0.569
8	Assam	Karimganj	1.146	0.783–1.439	2.85 ± 0.64	1.844	0.615	3	47.75	0.605
9	Arunachal Pradesh	Itanagar	0.092	0.061–0.133	1.37 ± 0.27	0.292	0.100	3	3.83	0.961
10	Assam	Chauribari	0.085	0.054–0.15	1.10 ± 0.21	0.346	0.120	3	3.54	0.951
11	Manipur	Senapati	0.065	0.036–0.106	1.45 ± 0.29	0.129	0.040	3	2.71	0.988
12	Tripura	Kumarghat	0.059	0.032–0.104	1.05 ± 0.22	0.135	0.050	3	2.46	0.987
13	Assam	Silchar	0.044	0.024–0.070	1.34 ± 0.27	0.115	0.040	3	1.83	0.990
14	Mizoram	Mammit	0.039	0.023–0.059	1.32 ± 0.24	1.654	0.550	3	1.63	0.647
15	Delhi	Lab susceptible (Pusa)	0.024	0.016–0.032	2.30 ± 0.41	2.113	0.700	3	1	0.547

Probit mortality and dose–response activity of 15 field populations collected from different states. LC_50_, median lethal concentration (concentration in mg/L that would kill 50% of the treated population). Fiducial limit is presented at the 95% confidence level. χ^2^, chi-square value at 95% confidence level; RR, resistance ratio (RR of a selected population = (LC_50_ of the selected population/LC_50_ of a susceptible population) × 100); H, heterogeneity; d.f, degrees of freedom (i.e., d.f = n−2, where n is the number of concentrations employed for bioassay); ‘*p*’ value at 0.05% level of significance.

**Table 3:** Antioxidant enzyme activities in field populations of *Rhyzopertha dominica*.

Sl. No	Location	Catalase (nmol/min/mg Protein)	Peroxidase (nmol/min/mg Protein)	SOD Activity (Inhibition Rate%)
1	Barhi	22.221 ± 0.208 ^bc^	2.583 ± 0.678 ^a^	85.759 ± 0.919 ^d^
2	Silchar	6.287 ± 0.113 ^a^	1.281 ± 0.320 ^a^	81.930 ± 0.403 ^ab^
3	Karimganj	23.833 ± 0.394 ^c^	13.487 ± 0.885 ^bc^	92.174 ± 0.382 ^g^
4	Hapur	11.104 ± 0.285 ^a^	1.999 ± 0.219 ^a^	86.975 ± 0.180 ^e^
5	Churaibari	35.223 ± 0.922 ^d^	2.104 ± 0.389 ^a^	83.004 ± 0.090 ^bc^
6	Kumarghat	59.061 ± 0.777 ^f^	1.805 ± 0.00 ^a^	83.456 ± 0.013 ^c^
7	Itanagar	32.991 ± 0.642 ^d^	10.430 ± 1.323 ^abc^	90.713 ± 0.093 ^f^
8	Kota	13.114 ± 0.212 ^ab^	25.278 ± 0.623 ^d^	98.531 ± 0.510 ^i^
9	Mummit	10.501 ± 0.108 ^a^	38.833 ± 0.987 ^e^	98.963 ± 0.299 ^i^
10	Patiala	7.701 ± 0.202 ^a^	183.720 ± 1.482 ^g^	98.778 ± 0.815 ^i^
11	Safidon	7.764 ± 0.094 ^a^	336.8 ± 10.024 ^h^	99.661 ± 0.068 ^i^
12	Karnal	8.771 ± 0.195 ^a^	63.520 ± 1.464 ^f^	99.050 ± 0.180 ^i^
13	Delhi	14.821 ± 0.444 ^abc^	18.015 ± 2.756 ^cd^	97.013 ± 0.339 ^h^
14	Senapati	46.337 ± 1.354 ^e^	9.014 ± 2.410 ^ab^	91.107 ± 0.068 ^fg^
15	Lab susceptible (Pusa)	320.13 ± 11.555 ^g^	5.350 ± 0.261 ^ab^	81.296 ± 0.358 ^a^

Specific activities of antioxidant enzymes in different field populations of *R. dominica*. The values of mean ± standard error followed by distinct letters are significantly different (*p* < 0.05). The treatment means were separated by Duncan’s multiple range test. Three replications of each enzyme (catalase, peroxidase, and SOD) were carried out to determine the activity presented in the table.

**Table 4:** Correlation and regression of resistance ratio vis-à-vis antioxidant enzymes in different field populations of *Rhyzopertha dominica*.

	Correlation with a Resistance Ratio	Coefficient (b)
Catalase	−0.707 **	−1.34 **
Peroxidase	0.632 **	0.205 **
Superoxide Dismutase	0.689 **	3.151 **

**, values at a 1% level of significance; ‘b’, regression coefficient.

## Data Availability

Data is contained within the article and Supplementary Materials.

## Supplementary Materials

The following supporting information can be downloaded at: https://www.mdpi.com/article/10.3390/antiox12020270/s1, Table S1: Parameters from the Biplot (Principal Component Analysis) viz., Eigenvalue, Cumulative Eigenvalue, % Variance, and Cumulative variance.
