# Supplementary material for: Differential Activities of Antioxidant Enzymes, Superoxide Dismutase, Peroxidase, and Catalase vis-à-vis Phosphine Resistance in Field Populations of Lesser Grain Borer (Rhyzopertha dominica) from India
Source: Antioxidants (Basel). 2023 Jan 25;12(2):270. doi: 10.3390/antiox12020270 (PMC9952823; doi:10.3390/antiox12020270)
Supplement: Supplementary file 1 [file antioxidants-12-00270-s001.zip › antioxidants-2012448-supplementary.pdf]

*Supplementary Materials*

**Table S1.** Parameters from the Biplot (Principal Component Analysis) *viz.*, Eigenvalue, Cumulative Eigenvalue, % Variance, and Cumulative variance.

| PC | Eigenvalue | % Variance | Cumulative Eigenvalue | Cumulative Variance |
|---|---|---|---|---|
| 1 | 2.45 | 61.37 | 2.45 | 61.37 |
| 2 | 0.82 | 20.42 | 3.27 | 81.79 |
| 3 | 0.39 | 9.69 | 3.66 | 91.48 |
| 4 | 0.34 | 8.52 | 4.00 | 100.00 |

This table briefs on the factors and their respective Eigenvalue and % cumulative variance chosen for the Principal Component Analysis (PCA). Respective % variance and Cumulative Eigenvalues are also shown in the table. Here, PC1 and PC2 were considered for our PCA analysis.
